# An urge to fill a knowledge void: Malaria parasites are rarely investigated in threatened species

**DOI:** 10.1371/journal.ppat.1008626

**Published:** 2020-07-02

**Authors:** María Cristina Carmona-Isunza, Sergio Ancona, Jordi Figuerola, Alejandro Gonzalez-Voyer, Josué Martínez de la Puente

**Affiliations:** 1 Departamento de Ecología Evolutiva, Instituto de Ecología, Universidad Nacional Autónoma de México, Ciudad de México, México; 2 Estación Biológica de Doñana (EBD-CSIC), Seville, Spain; 3 Ciber de Epidemiología y Salud Pública (CIBERESP), Seville, Spain; Children's Hospital of Philadelphia, UNITED STATES

## Summary

There is an increasing urgency in gaining greater understanding of the factors that affect vulnerability to extinction, given the current levels of species loss. Parasites often reduce host fitness and can thus exert an important influence on host population dynamics, exacerbating vulnerability to extinction. However, empirical support for the hypothesis that parasites can increase vulnerability to extinction is rare. A crucial factor limiting tests of the hypothesis is data availability. An extensive review of the literature revealed that common avian malaria and avian malaria–like haemosporidian parasites are seldom investigated in endangered birds. We stress the importance of assessing the occurrence of haemosporidians and other parasites in vertebrate species of high conservation concern.

## Why is it important to investigate parasites in threatened birds?

Accelerated biodiversity loss is one of the major challenges facing humanity. Around 14% of all bird species are threatened with extinction [1]. Although various factors play a role in the extinction of species, infectious diseases are often assumed to be a threat to population viability and thus a factor that contributes to the risk of extinction [2, 3]. A well-studied group of widespread parasites to which birds are vulnerable are haemosporidians of the genera *Plasmodium* (transmitted by mosquitoes), *Haemoproteus* (transmitted by *Culicoides* and hippoboscids), and *Leucocytozoon* (transmitted by black flies) that cause avian malaria and avian malaria–like diseases [4, 5]. Traditionally, impacts of haemosporidians on birds were assumed to be mild when hosts coevolved with these parasites. However, current evidence indicates that infection by haemosporidians involves reductions in bird survival and reproductive success and translates into population declines and increased risk of extinction, especially in immunologically naïve populations and when preepidemic population size is small [6]. The decline and extinction of native forest birds following introduction of *Plasmodium relictum* to Hawaii is an example of these detrimental effects [7]. Importantly, habitat alteration and current climatic changes may impact spreading and proliferation of vectors and thus rates of transmission of vector-borne diseases in nature [7, 8]. However, whether haemosporidians represent a risk for the persistence of wild bird populations is still an open question, and we argue that the data currently available do not allow for a robust test of this hypothesis.

Here, we provide evidence that the occurrence of haemosporidian parasites is poorly documented in most bird species in high extinction risk categories, according to the International Union for Conservation of Nature (IUCN) [1], and urge parasitologists, wildlife epidemiologists, ornithologists, and ecologists to study parasite prevalence and diversity in threatened species. Assessing the occurrence of haemosporidians in endangered birds is of utmost importance because parasites are often alleged to contribute to the risk of extinction [2], the percentage of the world’s bird species now considered as threatened is high and increasing [1], and blood parasites are common in wild bird populations [4] and can be easily screened using blood samples. The data generated in this line of research will be essential also for parasite conservation, as parasites may themselves be at risk of extinction nowadays [9], especially parasites specialized on threatened, rare, or geographically restricted hosts (e.g., the nearly extinct *Felicola isidoroi*, host-specific louse of the Iberian lynx [10]). Furthermore, future studies may provide valuable information for research on parasite–host dynamics and food security, as many haemosporidians that threaten wildlife may also threaten farmed birds [5].

## Data on the occurrence of blood parasites in the MalAvi database and the grey literature for threatened bird species

**Fig 1:**
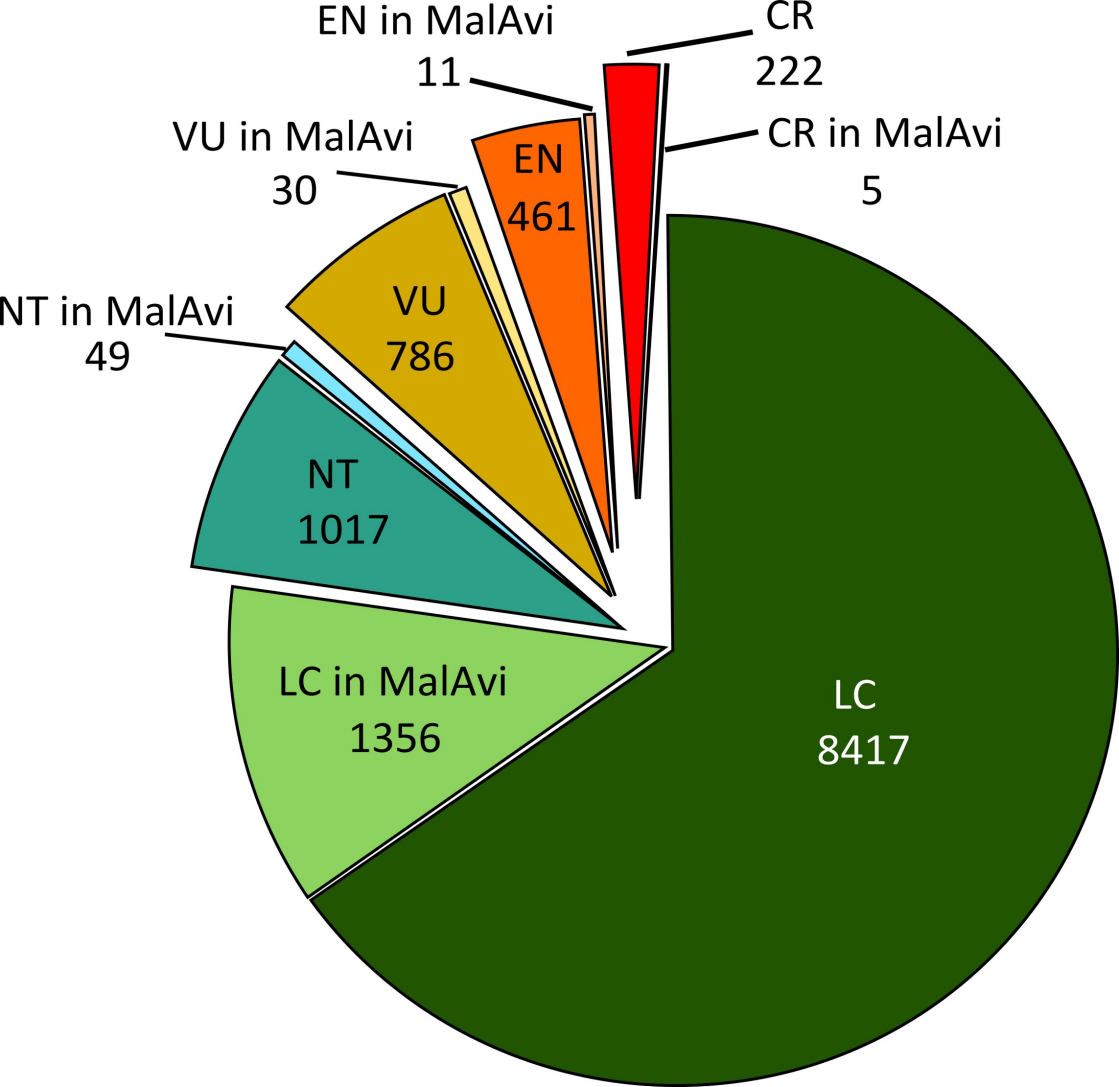
Number of avian species listed in each IUCN 2018 Red List Category and the subset of those that are present in MalAvi. CR, Critically Endangered; EN, Endangered, IUCN, International Union for Conservation of Nature; LC, Least Concern; NT, Near Threatened, VU, Vulnerable.

The IUCN Red List of Threatened Species includes 10,903 extant bird species, of which 8,417 are listed as Least Concern, 1,017 as Near Threatened, 786 as Vulnerable, 461 as Endangered and 222 as Critically Endangered [1] (Fig 1, S1 Data). For further details on this categorization, see [1]. We matched the list of bird species in IUCN with the list of host species included in MalAvi, the most comprehensive and continuously updated data set of avian malaria and malaria-like avian haemosporidians and their hosts [11]. To our surprise, the occurrence of blood parasites has been examined for only 3.82% of Vulnerable, 2.39% of Endangered, and 2.25% of Critically Endangered bird species (Fig 1, S1 Data). Numbers are higher for near threatened bird species (4.82%) and least concern (16.11%), but reporting of haemosporidians in birds listed in these two categories of lower risk is markedly low as well (Fig 1, S1 Data). Importantly, bird species listed as Least Concern are overrepresented in MalAvi, whereas bird species listed in the four other higher risk categories are underrepresented (chi-squared goodness of fit test: χ^2^ = 219.90; degrees of freedom = 4; *P* < 0.0001). It is important to note that MalAvi compiles only studies that have molecularly identified blood parasites based on the amplification of a fragment of the cytochrome *b* gene, and therefore all but two of the 388 included studies have been published since 2002 [11].

Vulnerable, Endangered and Critically Endangered bird species may be underrepresented in MalAvi because the occurrence of haemosporidians in these host species is rarely assessed or because studies not finding haemosporidians are not reported in MalAvi. Therefore, data potentially available in published papers and in the grey literature (e.g., technical reports, zoo reports, etc.) should also be explored. We performed an additional search by randomly choosing 50 bird species included in each of the three highest IUCN Red List Categories (Vulnerable, Endangered, and Critically Endangered). We searched for these 150 species in Google Scholar, using as search string each bird species’ scientific and common name and names of the three genera of parasites of interest: *Plasmodium* OR *Haemoproteus* OR *Leucocytozoon* (example: "*Amazona versicolor*" OR "St Lucia Amazon" OR "Saint Lucia Amazon" OR "Saint Lucia Parrot" OR "St. Lucia Amazon" OR "St. Lucia Parrot" AND *Plasmodium* OR *Haemoproteus* OR *Leucocytozoon*). This search returned reports of haemosporidians for only 5.3% of the species: two reports for two species listed as Vulnerable, four reports for four species listed as Endangered, and two reports for two species considered as Critically Endangered. These studies came from wild populations in which at least five individuals were sampled, for which PCR techniques were used to identify haemosporidians and for which prevalence could be estimated (see S2 Data). We limited our search to reports using PCR because this is the most reliable method for haemosporidian detection and identification [12]. We, therefore, conclude that the occurrence of haemosporidians is rarely investigated in birds facing high risk of extinction and consider it important to highlight the need of conducting more research on parasites in wild populations of highly threatened birds.

## Concluding remarks

Screening and/or reporting of avian malaria and malaria-like haemosporidians is rarely performed in bird species at high risk of extinction. Such bias in screening may have several explanations: (1) Critically endangered bird species are rare in the wild; (2) getting permits to collect samples from critically endangered or threatened bird species may be difficult for researchers, especially in protected areas; (3) threatened bird species could be difficult to study in the wild due to their biology, behavior or nesting sites (e.g., Puaiohi thrushes, *Myadestes palmeri* are endemic to Hawaii and breed in rocky cliffs); or (4) studies on endangered species do not include parasites among their priorities.

Regardless of the reasons, the lack of screening or reporting of parasites in endangered birds could result in the loss of irretrievable knowledge on the prevalence and diversity of parasites in the wild and their important role in ecological and evolutionary processes [13, 14]. Importantly, haemosporidians can be easily screened using blood samples, yet they are still understudied in threatened birds; we therefore conjecture that other parasites that are more difficult to study, such as helminths, might also be understudied in bird species at high risk of extinction. We anticipate that this awareness call will encourage research in haemosporidians and other groups of parasites, providing essential information to evaluate the relationship between parasitism and extinction risk for wild bird populations. The systematic screening of endangered species would help identify potentially new sources of risk for populations as well as unknown species of parasites. Moreover, efforts to collate data in comprehensive datasets like MalAvi are invaluable for comparative studies, and we call for the research community studying these parasites to continue their contribution in keeping such databases updated.

## Supporting information

S1 Data Database containing all avian species in IUCN and whether they appeared in MalAvi or not. (CSV)

S2 Data Database containing the random 150 species from the three highest IUCN Red List Categories (Vulnerable, Endangered and Critically Endangered) searched in Google Scholar for malaria-like haemosporidians. (XLSX)
